# Exposomic Fingerprint in the Development of Diseases: The Role of Free Radicals and Multiomics

**DOI:** 10.1155/2022/9851253

**Published:** 2022-03-20

**Authors:** Gordana Kocić, Andrej Veljković, Dušan Sokolović, Nataša Poklar Ulrih

**Affiliations:** ^1^Faculty of Medicine, University of Nis, 18000 Nis, Serbia; ^2^Department of Food Science and Technology, Biotechnical Faculty, University of Ljubljana, Jamnikarjeva 101, SI-1000 Ljubljana, Slovenia; ^3^The Centre of Excellence for Integrated Approaches in Chemistry and Biology of Proteins (CipKeBiP), Slovenia

The concept of the exposome refers to the totality of environmental exposures, including general or specific external, internal, and psychosocial factors, affecting the body from the embryonic period to the old age. The importance of exposome for the development of chronic diseases and multiorgan failure may account for about 70 to 90% of disease risks. It has been documented that the majority of important chronic diseases are likely to result from the combination of environmental exposures to infective, chemical, and physical stressors and their interaction with the human genome [1–4]. Most often, a number of different exposomic risk factors may act simultaneously and exert cumulative effect.

When trying to understand the factors responsible for the individual sensitivity to the development of malignant and chronic diseases, the emphasis has been placed on individual genetic variations due to single-nucleotide polymorphism (SNP) and their proteome-related response. They can be defined as genetic exposure susceptibility to disease development. Polymorphic variants in oxidative stress enzymes may explain the interindividual variability in response to chronic disease, tumor development, and tumor therapy.

Among specific external exposures, the infectious agents, especially viruses, environmental pollutants, drugs, diet, lifestyle factors (smoking, alcohol abuse), and medical interventions (supplemental oxygen ventilation and high flow oxygen therapy of critically ill patients), may affect cell and tissue systems, due to a deregulated free radical production and their neutralization [5]. The balance between the exposome and endogenous circulating hormones, body composition, aging, inflammation, wider social, economic, and psychological influences may affect the host individual response and the disease outcome.

The objective of the special issue is to provide molecular mechanisms related to how specific environmental factors provoke reactive oxygen species (ROS) production, and how ROS, as a part of environmental exposure or internal production, can affect human health. It includes articles related to the role of free radicals and multiomics in describing exposomic marker and other biomarkers of a disease. The originality lies in the ability to distinguish and recognize the earliest and innovative exposomic biomarkers accurately, the earliest genetic predisposition markers, and new therapeutic influences in disease diagnostics, prevention, and treatment. The articles may be clustered concerning specific type(s) of diseases and to specific environmental factors. All of the abovementioned exposomic factors are shown in Figure 1.

Devastating effects of viral infections, particularly the COVID-19 (SARS-CoV-2) pandemic and HCV infection, may belong to the most important external exposures at the moment. Four of eight published articles refer to viral infections. They are related to a deeper explanation of molecular mechanisms associated with late consequences and serious organ damage. The authors Zdravković M et al. and Popadić et al. in their collaborating studies nested from more than 1000 COVID-19 patients per month. They emphasized the importance of the appropriate risk stratification in 460 COVID-19 patients with a higher risk of poor clinical outcomes at admission to the ICU, under the specific (prooxidative) conditions demanding high oxygen flow. The clinical outcome of the disease includes acute respiratory distress syndrome, superinfection, shock, acute heart, liver, and kidney injury, evaluated also by specific laboratory biomarkers. The use of AIDA score has been defined as a reliable tool for delivering the appropriate therapy on time. It was developed by combining significant variables from the multivariate logistic regression analysis including serum albumin, interleukin-6, and D-dimer, accompanied by age. They also developed and validated a multivariable predictive model for mortality of COVID-19 patients at admission to ICU, in relation to respiratory status (development of acute respiratory distress syndrome ARDS) as the most important clinical outcome. The appearance of ARDS was considered in relation to invasive or noninvasive mechanical ventilation and high flow oxygen therapy. In the final multivariate analysis, serum albumin (below 33 g/L), interleukin-6 (above 72 pg/mL), and D-dimer, accompanied by age and CT severity score as parts of univariate analysis, were marked as independent predictors of mortality. These predictors have been referred to the three most probable pathophysiological mechanisms of a lethal outcome, septic infections associated with septic shock, procoagulable state, procoagulant state associated with micro- and macrothrombosis, and cytokine storm proceeded to multiorgan failure. The COVID-19 research-related articles were followed by a research of Cekerevac et al. This is the first study where it documented the level of circulating oxidative stress parameters as predictors in the disease severity and mortality. In their prospective cross-sectional study, they reported novel information about potential molecular mechanisms during the different degree of COVID-19 in adult patients, due to the infiltration of neutrophils, a marked elevation of proinflammatory cytokines, and cytokine storm association with elevated levels of superoxide anion radicals. They may act as significant contributors to the disease progress, severity, and mortality. Moreover, by using a linear regression model, they documented that hypertension, anosmia, ageusia, the O_2_^−^level, and the duration at the ICU may be predictors of severity of COVID-19 (SARS-CoV-2) disease in a group of 127 patients. In order to evaluate the influence of severity of the disease, all patients were divided into a group with a mild form of COVID-19 (mild symptoms up to mild pneumonia, with moderate COVID-19 form (dyspnea, hypoxia, or less than 50% lung involvement on imaging) and a group of patients with a severe COVID-19 (severe respiratory failure, high flow oxygen therapy, mechanical ventilation, sepsis, or multiorgan system dysfunction). Besides the evaluation of standard laboratory markers, the concentration of prooxidative markers (superoxide anion radical (O_2_^−^), hydrogen peroxide (H_2_O_2_), nitric oxide (NO^−^), and lipid peroxidation (TBARS)) and antioxidative markers (catalase (CAT), superoxide dismutase (SOD), and reduced glutathione GSH) were determined in blood plasma and lysate.

The association of chronic HCV infection-specific genotype and viral load association with increased oxidative stress has been emphasized by a comprehensive study by Đorđevic et al. The intensity of oxidative stress may be a detrimental factor in liver injury and may determine the severity of the disease. In their case-control study, which involved 52 HCV patients and 50 control healthy patients, they demonstrated the intensity of oxidative stress (level of lipid peroxidation TBARS and protein oxidative modification AOPP) and decreased antioxidative defense (catalase activity) as a detrimental factor in liver injury and severity of the disease. The cells responsible for ROS production and liberation are hepatocytes, Kupffer cells (resident macrophages), inflammatory cells, hepatic stellate cells (HSCs), and other immune effector cells. The values of oxidative stress parameters (TBARS and AOPP) and catalase activity in patients infected with different HCV genotypes revealed that HCV1b patients were more likely to have a higher TBARS compared to others, HCV 3 patients were more likely to have a higher AOPP level, while patients infected with HCV1a were more likely to have a low catalase activity. A positive correlation was found between virus genome copy concentration and AOPP, while a high level of HCV viral load was more likely to have a higher TBARS. In a gender-based comparison, a significantly higher level of AOPP was reported in female patients. The results obtained confirmed the existence of imbalance between the ROS production and antioxidative defense system in HCV-infected patients. Since oxidative stress may have a profound influence on disease progression, fibrosis, and carcinogenesis, our results may meet the aspirations of mandatory introduction of antioxidants as early HCV therapy to counteract ROS consequences.

In in the cohort of 292 participants of adult population, Klisic et al. highlighted the role of internal exposome as potential factors specific to the individual physiology, age, and body morphology and their relation to oxidative stress markers. They examined a prooxidant-antioxidant balance (PAB)) and the marker of antioxidant defense capacity (total sulphydryl groups (tSHG)), their ratio (PAB/tSHG) and their relationship with different cardiometabolic risk factors (waist-to-height ratio, body mass index, visceral adiposity index, and lipid accumulation products). They reported tSHG and PAB/tSHG correlation with lipid parameters (HDL-c and TG) and lipid indices (VAI and LAP); HDL-c showed negative and TG, VAI, and LAP positive independent associations and predictions. To assess the associations of clinical markers with tSHG, PAB levels and PAB/tSHG index univariate and multivariate ordinal regression analyses were applied. By using principal component analysis (PCA), various cardiometabolic risk parameters produced scores for significant factors, which were used in the subsequent binary logistic regression analysis to estimate the predictive potency of the factors towards the highest PAB and tSHG values. In that way, obesity-renal function-related factor predicted both high PAB and low tSHG, while obesity-dyslipidemia-related factor predicted significantly high tSHG values. The authors concluded that unfavorable cardiometabolic profile was associated with higher tSHG values.

Durand et al. addressed the importance of oxidative stress as a pathogenetic key for development of nonalcoholic fatty liver disease (NAFLD). The severity of hepatic damage depends on whether simple fat accumulation (steatosis), nonalcoholic steatohepatitis (NASH), and hepatic fibrosis are developed. The lipid composition of mitochondrial membranes, presumably the composition of PE and CL is of crucial importance in maintaining mitochondrial structure and function in the assembly and activity of respiratory chain complexes III and IV and supercomplex formation. Accordingly, in the transport of electrons from complex I to ubiquinone, the oxidized state of coenzyme Q (CoQ) depends on the composition of lipophilic environment, i.e., cardiolipins (CLs). Their experimental model of NAFLD has been developed to integrate specific “omics” signature (lipidomics) and oxidative stress markers specifically adapted for mitochondrial liver cell fraction. Moreover, the mRNA expression levels for the key transcription factors for lipid synthesis, sterol regulatory element-binding transcription factor 1 (*Srebf1*), nuclear receptor peroxisome proliferator-activated receptor *α* (*Pparα*) and inflammation, Toll-like receptor 9 (*Tlr9*), and tumor necrosis factor (*Tnf-α*) were evaluated. The cardiolipin and CoQ antioxidant function impairment precede fibrosis, emphasizing a causal relationship with NAFLD development and progression. The optimal structure of mitochondrial lipids may represent a new therapeutic intervention in nonalcoholic fatty liver disease (NAFLD) prevention strategy.

It was documented that cancer develops from a combination of exposomic factors influencing specific susceptibility genes and family history. Among exposomic factors, there are specific external exposures and body-related internal exposome (Figure 1). The clear-cell renal cell carcinoma (ccRCC) belongs to types of carcinomas associated with Keap1/Nrf2 (Kelch-like ECH-associated protein 1/nuclear factor (erythroid-derived 2)-like2) pathway alterations. The authors Mihailovic et al. highlighted the relation between the reactive oxygen species and electrophiles and the activation of specific adaptive cytoprotective response, including changes in the Keap1/Nrf2 pathway. The enzymes encoded by Nrf2 target genes are glutathione S-transferases (GST), superoxide dismutase (SOD2), and glutathione peroxidase (GPX). The interaction between GSTP1: JNK1 may reveal the functional link between the upregulated GSTP1 and malignant phenotype. At the same time, the reaction catalyzed by mitochondrial SOD2 releases H_2_O_2_, which may act as the signaling molecule in cell proliferation, differentiation, and migration. It may also induce the activation of AMP-activated kinase activating glycolysis, where the energy production is essential for malignant cell survival. The antioxidant enzyme, glutathione peroxidase (GPX), catalyzes the reduction of H_2_O_2_. The single-nucleotide polymorphisms of Nrf2 gene and genes encoding GSTP1, SOD2, and GPX1 may change the expression of specific protein or affect the activity of synthesized proteins. The authors investigated the effect of specific *Nrf2*, *SOD2*, and *GPX1* gene variants and *GSTP1ABCD* haplotype on ccRCC risk and the prognosis in 223 ccRCC patients and 336 matched controls by PCR-CTTP and qPCR. Haplotype analysis revealed a significant risk of ccRCC development in carriers of the *GSTP1C* haplotype, while GSTP1 variant affected the overall survival in ccRCC patients. The increased ccRCC susceptibility was observed among carriers of individual variant genotypes of *SOD2* and *GSTP1* and *Nrf2*. Moreover, the analysis of *GSTP1ABCD* haplotype revealed significant risk of ccRCC development and the overall survival in patients with ccRCC. This interaction is summarized in Figure 2.

Jerotic Dj et al. reported in an experimental study a nowel mechanism of glutathione S-transferase M1 (GSTM1) downregulation influence on increased oxidative stress and inflammation in endothelial cells in uremic conditions. The also reported that the deletion polymorphism of glutathione S-transferase M1 (GSTM1), a phase II detoxification and antioxidant enzyme, increased susceptibility to end-stage renal disease (ESRD), as well as the development of cardiovascular diseases (CVD) among ESRD patients and their shorter cardiovascular survival. When human umbilical vein endothelial cells (HUVECs) were exposed to uremic serum, they exhibited impaired redox balance, associated with enhanced lipid peroxidation and decreased antioxidant enzyme activities, expression of a series of inflammatory cytokines including retinol-binding protein 4 (RBP4), regulated upon activation, normal T cell expressed and secreted (RANTES), C-reactive protein (CRP), angiogenin, dickkopf-1 (Dkk-1), and platelet factor 4 (PF4). In order to obtain *GSTM1-null* genotype, with no GSTM1 protein expression, HUVECs were transfected with GSTM1 small interfering RNA (siRNA). The authors documented that *GSTM1-null* genotype exposed to uremic serum led to the upregulation of monocyte chemoattractant protein-1 (MCP-1), intracellular and vascular cell adhesion molecules (ICAM-1 and VCAM-1). Very interesting was the finding on the increased levels of serum ICAM-1 and VCAM-1 (sICAM-1 and sVCAM-1) in ESRD patients lacking GSTM1, in comparison with patients with the *GSTM1-active* genotype. A novel function of endothelial GSTM1 in the regulation of monocyte migration and adhesion, through its role in the upregulation of MCP-1, might be relevant as a potential therapy target.

In summary, this special issue is compatible and consistent with the research topic requirements, delineated in the aim and description proposed for this special issue:

- to perform translational studies focused on specific external exposures (viruses COVID-19 and HCV), by using human blood samples
- to create *in vivo* experimental animal models for the examination of the role of free radical-induced chronic diseases in relation to proteome and lipidome signature (NAFLD)
- to perform *in vitro* cell culture models to explore a role of free radicals in chronic diseases (HUVEC)
- to identify and characterize potential cell signaling pathways sensing specific external exposures and body-related internal exposome in chronic disease and tumor development (ccRCC and cardiometabolic syndrome)
- to identify and characterize the novel regulatory pathways connecting redox-sensitive signaling enzymes to cell transcriptome and proteome and small interfering RNA silencing connection with redox enzymes (*GSTM1-null* genotype)

## Figures and Tables

**Figure 1:**
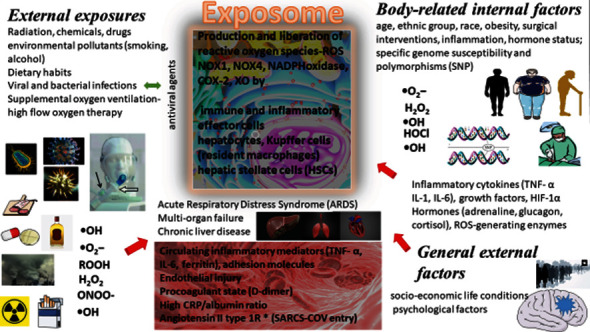
The concept of the exposome and its relation to cell injury and laboratory markers.

**Figure 2:**
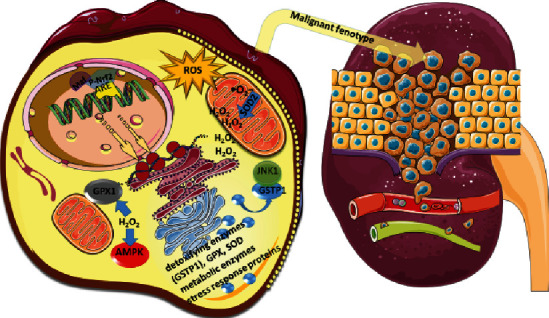
Relation between p-Nrf2 and expression pattern of detoxifying enzymes (created according to the results referred by Mihailovic et al.).
